# Platinum

**Published:** 1860-10

**Authors:** 


*Platinum.*—It is a proof of great advancement in the department of metallurgy that this metal is now cast in iron ingot moulds, by the process of St. Claire Deville and Debray. The process is described in our present number. B.

